# Left Atrial Appendage Morphology in Patients with Suspected Cardiogenic Stroke without Known Atrial Fibrillation

**DOI:** 10.1371/journal.pone.0118822

**Published:** 2015-03-09

**Authors:** Miika Korhonen, Antti Muuronen, Otso Arponen, Pirjo Mustonen, Marja Hedman, Pekka Jäkälä, Ritva Vanninen, Mikko Taina

**Affiliations:** 1 Department of Clinical Radiology, Kuopio University Hospital, Kuopio, Finland; 2 Unit of Radiology, Institute of Clinical Medicine, University of Eastern Finland, Kuopio, Finland; 3 Department of Cardiology, Keski-Suomi Central Hospital, Jyväskylä, Finland; 4 Heart Center, Kuopio University Hospital, Kuopio, Finland; 5 NeuroCenter, Kuopio University Hospital, Kuopio, Finland; 6 Unit of Neurology, Institute of Clinical Medicine, University of Eastern Finland, Kuopio, Finland; National Cerebral and Cardiovascular Center, JAPAN

## Abstract

The left atrial appendage (LAA) is the typical origin for intracardiac thrombus formation. Whether LAA morphology is associated with increased stroke/TIA risk is controversial and, if it does, which morphological type most predisposes to thrombus formation. We assessed LAA morphology in stroke patients with cryptogenic or suspected cardiogenic etiology and in age- and gender-matched healthy controls. LAA morphology and volume were analyzed by cardiac computed tomography in 111 patients (74 males; mean age 60 ± 11 years) with acute ischemic stroke of cryptogenic or suspected cardiogenic etiology other than known atrial fibrillation (AF). A subgroup of 40 patients was compared to an age- and gender-matched control group of 40 healthy individuals (21 males in each; mean age 54 ± 9 years). LAA was classified into four morphology types (Cactus, ChickenWing, WindSock, CauliFlower) modified with a quantitative qualifier. The proportions of LAA morphology types in the main stroke group, matched stroke subgroup, and control group were as follows: Cactus (9.0%, 5.0%, 20.0%), ChickenWing (23.4%, 37.5%, 10.0%), WindSock (47.7%, 35.0%, 67.5%), and CauliFlower (19.8%, 22.5%, 2.5%). The distribution of morphology types differed significantly (P<0.001) between the matched stroke subgroup and control group. The proportion of single-lobed LAA was significantly higher (P<0.001) in the matched stroke subgroup (55%) than the control group (6%). LAA volumes were significantly larger (P<0.001) in both stroke study groups compared to controls patients. To conclude, LAA morphology differed significantly between stroke patients and controls, and single-lobed LAAs were overrepresented and LAA volume was larger in patients with acute ischemic stroke of cryptogenic or suspected cardiogenic etiology.

## Introduction

Stroke is the leading cause of long-term disability and a major consumer of health care resources in both Western society and worldwide [1]. Atrial fibrillation (AF), either chronic or paroxysmal (PAF), is the most common causes of cardioembolic stroke [2]. Over 90% of cardiac thrombi are formed in the left atrial appendage (LAA) in patients with non-rheumatic AF [3]. Defining the direct source of embolism is often difficult and requires various imaging modalities [4] and rhythm monitoring. Three-week telemetry has revealed PAF in almost 20% of cryptogenic stroke cases, whereas only 4–8% of AF has been detected with 48-hour monitoring [5–6]. AF and PAF may enlarge the LAA [7–8], and remodeled LAA structures may predispose an individual to atrial arrhythmias [9]. Certain LAA morphology type may affect rheology, flow velocity and coagulation tendency [10]. To avoid stroke recurrence, the recognition of morphological signs indicative of latent PAF and susceptibility to thrombus formation would be helpful.

The aim of the present study was to assess whether certain LAA morphologies detected in cardiac computed tomography (cCT) are associated with acute ischemic stroke of cryptogenic or suspected cardiogenic etiology other than known AF.

## Materials and Methods

The Kuopio University Hospital Research Ethics Board approved the study (N:o 82/2004). Prior to participation in the study, written informed consent was obtained from each patient or their legally authorized representative if a patient was unable to provide consent due to impaired mental or physical function caused by stroke. All clinical investigations have been conducted according to the principles expressed in the Declaration of Helsinki.

### Stroke/TIA Patients and Control Population

Patients with acute stroke/TIA admitted to our university hospital between March 2005 and November 2009 were evaluated as candidates for the EmbodeteCT study [4,11]. Altogether 162 consecutive patients who were evaluated by neurologists and had acute ischemic stroke of cryptogenic or suspected cardiogenic etiology without previously reported AF and without AF diagnosed at the time of enrollment were recruited. After enrollment, ambulatory 24-h Holter ECG was performed. A total of 51 patients were excluded after recruitment for the following reasons: confirmed small vessel disease or carotid/vertebral artery stenosis (n = 38), cCT image quality inappropriate for LAA morphology analysis (n = 4), ECG synchronization failed (n = 3), contrast media injection failed (n = 2) or was contraindicated due to renal insufficiency (n = 1), and refusal to participate despite previously giving informed consent (n = 3). The remaining 111 patients (74 males; mean age 60.4 ± 11.2 years) formed the main stroke group for the study. Forty patients (21 males; mean age 53.9 ± 9.3 years) were selected to form a matched stroke subgroup for pair-wise comparisons with the healthy controls. In addition, the main stroke group was compared to healthy controls.

The control population was selected as follows. A total of 243 consecutive patients underwent coronary CT angiography (CTA) for exclusion of coronary artery disease between December 2008 and July 2011. After excluding patients with coronary artery stenosis ≥50%, AF, hypertension, renal insufficiency, malignancies, or any neurological diagnosis, including any symptoms indicating stroke or TIA, 124 suitable candidates were available for pair-wise comparisons. No additional 24-h Holter ECG monitoring was performed. Forty individuals (21 males; mean age 53.8±9.0 years) were used to form the control group and to create age- and gender-matched pairs with the matched stroke subgroup.

### CT Imaging and Data Assessment

Contrast-enhanced cCT was performed with a 16-slice (113 patients) or 64-slice (36 patients, 40 control subjects) scanner (Somatom Sensation 16 and Somatom Definition AS; Siemens Medical Solutions, Forchheim, Germany). Control subjects with initial heart rates > 65 beats per minute received 5–20 mg metoprolol intravenously before examination. In stroke patients, the aortic arch and cervical and intracranial arteries were scanned first, immediately followed by the ascending aorta and heart. Cardiac imaging was performed during mid-diastole, with the entire LAA fully opacified with contrast media in all study subjects. In the 16-slice scanner, collimation was 16×0.75 mm, rotation time 0.42 s, and tube potential 120 kV; the current was set to 500 mAs for the first 80 patients and reduced to 250 mAs thereafter. In the 64-slice scanner, collimation was 64×0.6 mm, rotation time 0.33 s, and tube potential 120 kV; the reference current was set using commercially available tube current modulation software (CAREDose4D, Siemens Medical Solutions). In coronary CTA, collimation was 64×0.75 mm, rotation time 0.17 s, tube potential 120 kV, and tube current 327 mAs. The radiation dose per patient was 10.0±3.5 mSv in patients with stroke and 7.9±2.3 mSv in patients with coronary CTA. Mid-diastolic 0.75 to 1.0-mm-thick slices with 20–25% overlap were reconstructed.

### Assessment of LAA Morphology and Volume

**Fig 1:**
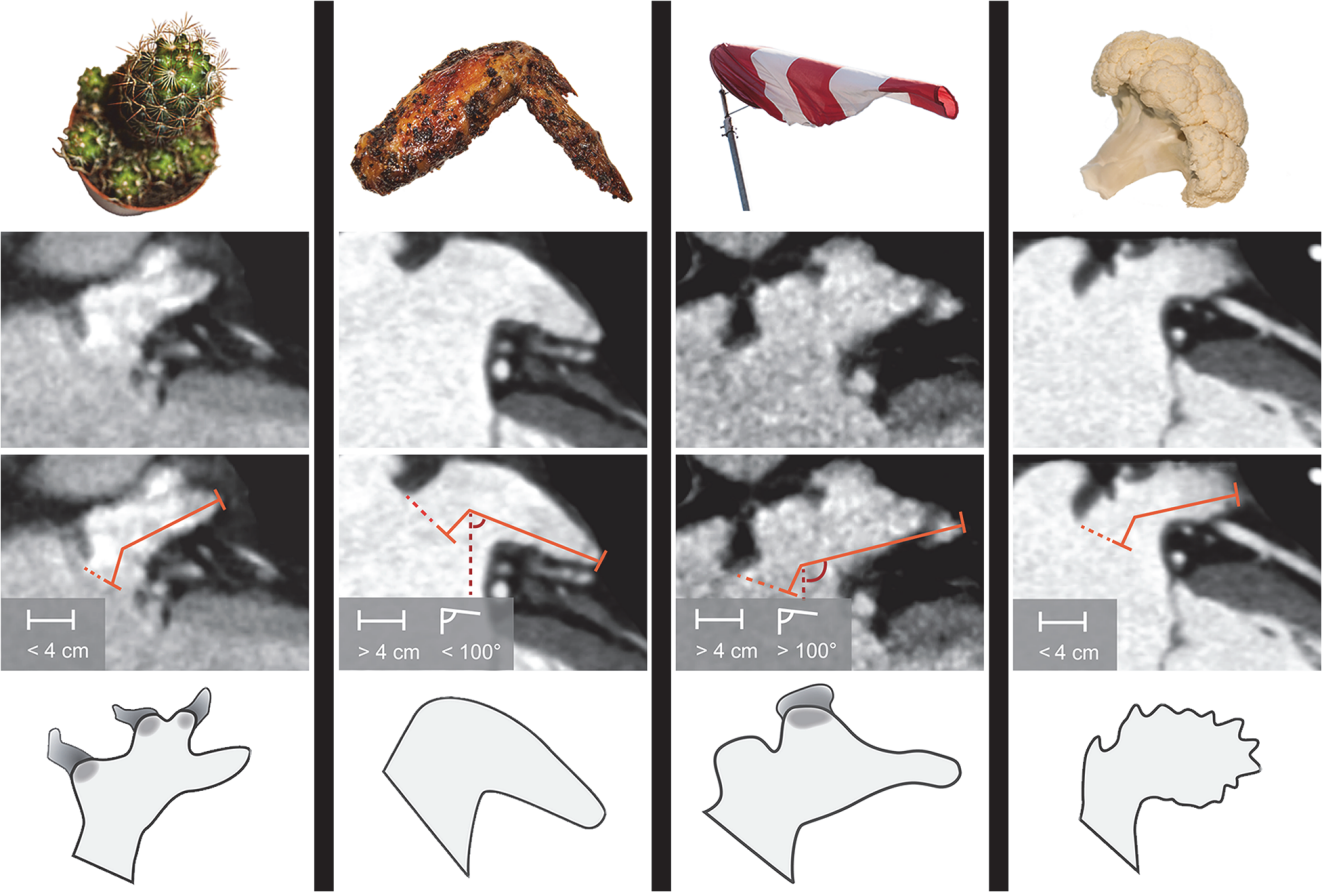
LAA morphology types based on Wang’s classification with Kimura’s quantitative limits. LAA length was measured from the orifice area (dashed orange line) to the farthest point of the LAA via the center of the main lobe. The bend angle was measured with an imaginary vertical line (red dashed line) and a line between the main lobe and the farthest point of the LAA. Cactus has a dominant central lobe, one or more secondary lobes, and total length less than 4 cm. ChickenWing has only one lobe, total length more than 4 cm, and a bend angle less than 100°. WindSock has one dominant lobe with several secondary, or even tertiary lobes, total length more than 4 cm, and a bend angle of over 100°. CauliFlower has a total length less than 4 cm and complex internal structures.

Quantitative image analysis was performed on an IDS5 diagnostic workstation (version 10.2P4; Sectra Imtec, Linköping, Sweden). Three independent observers blinded to collateral readings and all patient history visually analyzed LAA morphology according to the classifications of Wang et al [12] and Kimura et al [13] by assigning the LAAs into four classes: Cactus, ChickenWing, WindSock, and CauliFlower (Fig. 1). The Cactus is characterized by a dominant central lobe (length < 40 mm) and has one or more secondary lobes; ChickenWing has only one lobe (length > 40 mm) with bending less than 100° in the proximal part of the LAA; WindSock has one dominant lobe (length > 40 mm) with several secondary, or even tertiary lobes, with bending over 100°; CauliFlower is characterized by length less than 40 mm and with complex internal structures. All observers defined the following measurements: shortest distance to the LAA orifice from the mitral annulus (in millimeters), the number of LAA lobes (1, 2, or ≥3), amount of intra-LAA trabeculation (mild, moderate, or severe), and orientation of the LAA tip (1 = up, 2 = horizontal, 3 = down). After individual assessments, a consensus was reached based on the visual and quantitative observations. To assess reproducibility of the classification of LAA morphology two observers re-evaluated altogether 40 randomly chosen stroke patients from the main stroke group. In these patients, morphologies were analyzed in both arterial and venous phases.

LAA volumes were calculated using Simpson’s method by multiplying each manually traced area by the section thickness and summing the volumes of the separate sections [14]. Based on the volume measurements in the control population, the upper threshold for normal body surface area-adjusted (BSA-adjusted) LAA volume was defined as 5.6 mL/m^2^ [11]. The LAA volumes are presented as unadjusted values or values adjusted for height and BSA. BSA was calculated using Mosteller’s formula [15].

### Statistical Analysis

Continuous variables are presented as mean ±SD and categorical variables as absolute values and percentages. Significance was set to *P*<0.05 and high significance to *P*<0.001. Cohen’s kappa was used to test reproducibility of the assessments. Spearman’s correlation coefficient was used to investigate the associations between continuous variables and Fischer’s χ^2^ test to investigate nominal variables. Bonferroni correction was used to counteract the problem of multiple comparisons in four LAA morphologies, which set the significance at *P*<0.0125. Based on the Kolmogorov-Smirnov test, the Student’s *t*-test and Mann Whitney U test were used to compare dichotomized groups with normally distributed or abnormally distributed variables, respectively. An enlarged LAA was defined as LAA volume exceeding the mean volume in the control population by at least two standard deviations (SDs). Data were analyzed using SPSS for Windows (version 19, 1989–2010 SPSS Inc., Chicago, USA).

## Results

The clinical characteristics of the patients in the main stroke group, matched stroke subgroup and control group are presented in Table 1. The patients in the matched stroke subgroup were significantly more obese, had larger BSA, and more hypertension.

**Table 1:** Clinical Characteristics of the Main Stroke Group (n = 111), the Matched Stroke Subgroup (n = 40) and Control Group (n = 40).

Characteristic	Main stroke group	Matched stroke subgroup	Control group	*P*-value ^a^
Age, years	60.4 ± 11.2	53.9 ± 9.3	53.8 ± 9.0	ns
Males	74 (66.7)	21 (52.5)	21 (52.5)	ns
Body mass index, kg/m^2^	27.8 ± 4.4	28.7 ± 4.8	25.3 ± 4.1	0.002
Body surface area, m^2^	2.0 ± 0.2	2.0 ± 0.2	1.8 ± 0.2	0.033
Caucasian	111 (100)	40 (100.0)	40 (100.0)	ns
Hypertension	63 (56.8)	16 (40.0)	0 (0.0)	<0.001
Dyslipidemia	39 (35.1)	14 (35.0)	16 (40.0)	ns
Diabetes	12 (10.8)	0 (0.0)	0 (0.0)	ns
Smokers	27 (24.3)	9 (22.5)	1 (2.5)	0.003
Left ventricle dysfunction, n (%)	0 (0.0)	0 (0.0)	0 (0.0)	ns
AF or AFl on 24- hour ambulatory Holter ECG	17 (15.3)	0 (0.0)	0 (0)	ns
Left ventricle ejection fraction, %	63.6 ± 10.4 ^b^	66.0 ± 10.2 ^c^	63.7 ± 7.7	ns
Prior myocardial infarction	10 (9.0)	2 (5.0)	0 (0.0)	ns
Prior stroke	19 (17.1)	5 (12.5)	0 (0.0)	0.021
Left atrium volume on CT, mL	95.3 ± 31.2	85.5 ± 21.1	59.8 ± 15.3	<0.001
Left atrium volume on CT adjusted for height, mL/m	55.6 ± 17.6	50.5 ± 12.6	35.1 ± 8.0	<0.001
Left atrium volume on CT adjusted for BSA, mL/m^2^	48.7 ± 15.6	43.9 ± 10.9	32.2 ± 6.7	<0.001
Left atrial appendage volume on CT, mL	12.6 ± 5.2	10.2 ± 3.3	6.2 ± 1.9	<0.001
Left atrial appendage volume on CT adjusted for height, mL/m	7.4 ± 3.0	6.5 ± 2.2	3.7 ± 1.1	<0.001
Left atrial appendage volume on CT adjusted for BSA, mL/m^2^	6.5 ± 2.7	5.7 ± 2.0	3.4 ± 1.1	<0.001
LAA thrombus in TEE and CT	6 (5.4)	0 (0)	0 (0.0)	ns
LAA peak flow velocity, cm/s	0.65 ± 0.6 ^d^	0.64 ± 0.4 ^e^	NA	
Patent foramen ovale	11 (11.2) ^f^	5 (13.2) ^g^	NA	
Prosthetic aortic or mitral valve	0 (0)	0 (0)	0 (0)	ns
Aortic stenosis	1 (0.9)	0 (0)	0 (0)	ns
Mitral stenosis	0 (0)	0 (0)	0 (0)	ns
Mild or moderate mitral regurgitation	32 (35.6) ^h^	9 (30.0) ^i^	NA	
Moderately severe or severe mitral regurgitation	1 (1.1) ^h^	0 (0) ^i^	NA	

Data are given as n (%) or mean ± SDAF = Atrial Fibrillation; AFl = Atrial Flutter; CT = Computed Tomography; ECG = Electrocardiography; NA = Not assessed; ns = not significant; TEE = Transesophageal echocardiogram

^a^ Statistical significance between matched stroke subgroup and control group

^b^ n = 109

^c^ n = 37

^d^ n = 79

^e^ n = 31

^f^ n = 98

^g^ n = 38

^h^ n = 90

^i^ n = 30

**Fig 2:**
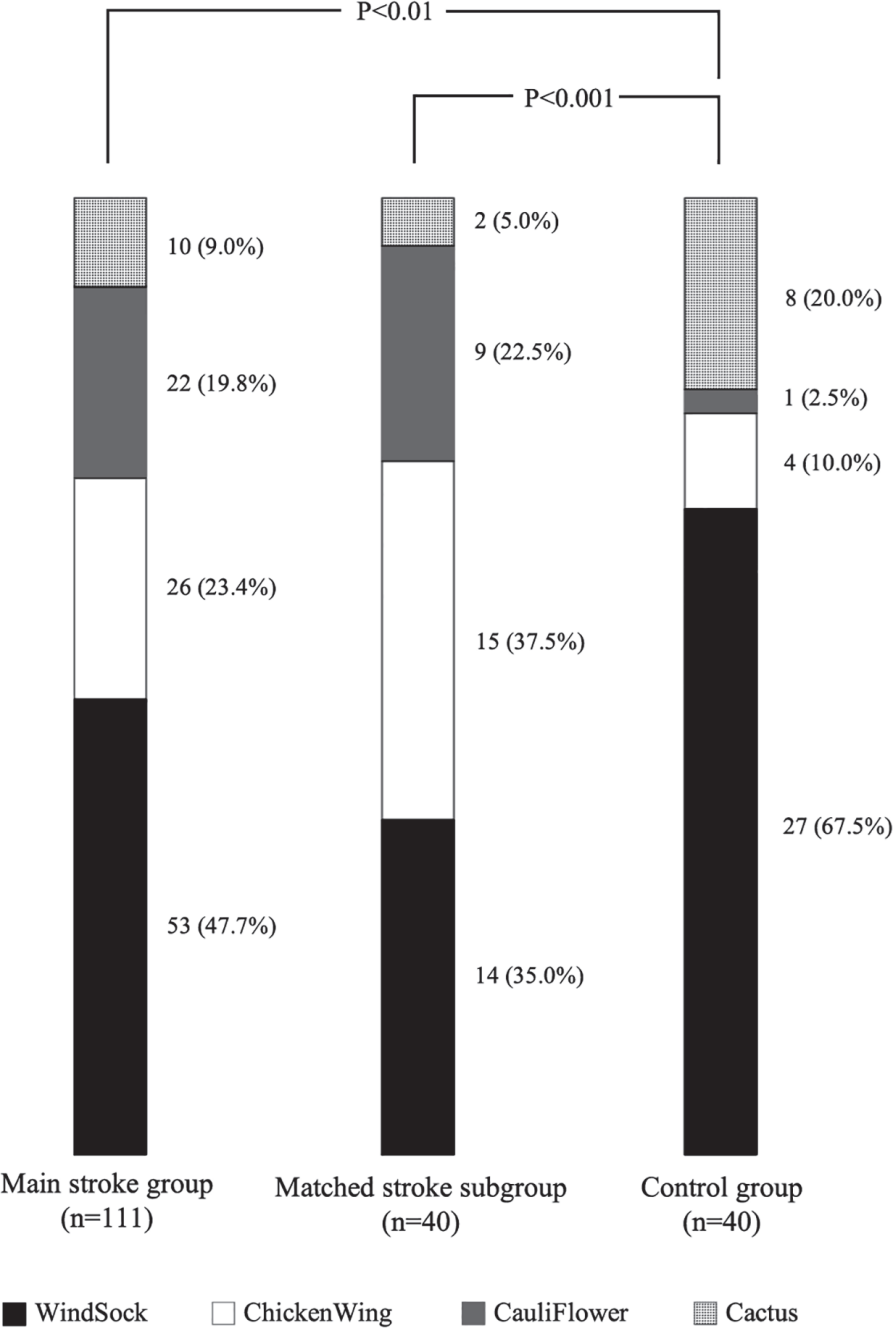
Prevalence of LAA morphologies among the three study groups.

The distribution of morphology types is presented in Fig. 2; a significant difference was found between the main stroke group and the control group (*P*<0.01). The difference was highly significant (*P*<0.001) between the age- and gender-matched stroke subgroup and the control group. After Bonferroni correction, ChickenWing morphology was significantly more frequent in the matched stroke subgroup (*P* = 0.008), whereas the proportion of WindSock was decreased (*P* = 0.007) compared to controls.

**Fig 3:**
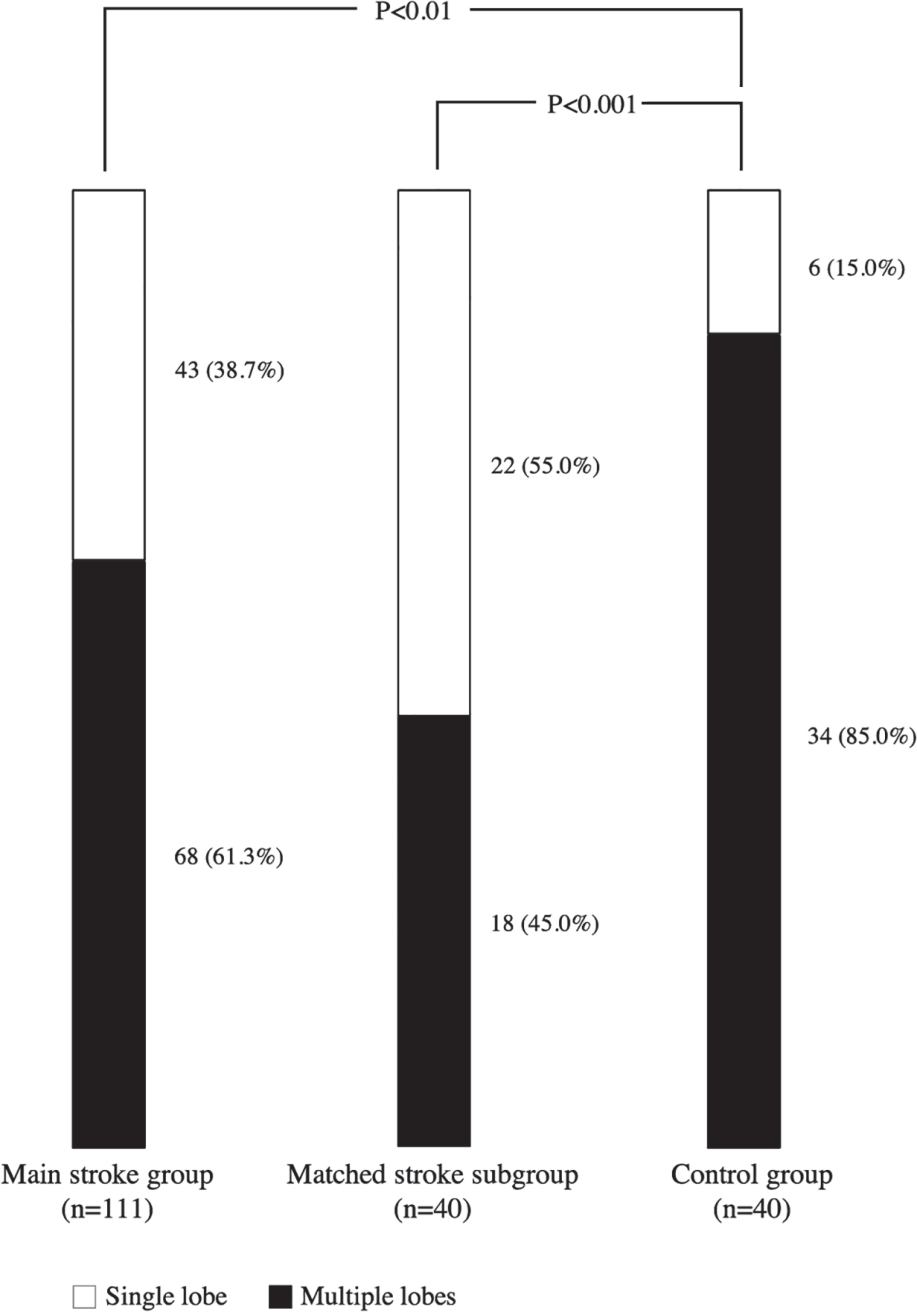
Prevalence of single-lobed LAA and multiple lobe (≥ 2) LAA in the three study groups.

Single-lobed LAAs were significantly more frequent in the main stroke group (39% vs. 15%; *P*<0.01), and even more frequent in the matched stroke subgroup (55% vs. 15%; *P*<0.001), compared to controls (Fig. 3). LAA volumes were larger in the main stroke group (6.5±2.7 mL/m^2^; *P*<0.001) and matched stroke subgroup (5.7±2.0 mL/m^2^; *P*<0.001) compared to controls (3.4±1.1 mL/m^2^). Also, the LAA opening height was larger (*P*<0.05) in the matched stroke group compared to controls. No differences were found in terms of LAA trabeculations or tip orientation. In addition, no significant difference was found between the main stroke group and matched stroke subgroup in regards to LAA morphology, LAA volume and number of lobes, opening height, trabeculation, or orientation of the tip.

Table 2 shows the associations between various morphology types and other LAA variables. LAA volumes varied significantly between LAA morphology types, with the WindSock type being significantly larger and the CauliFlower type significantly smaller than the other types.

**Table 2:** Comparison of Morphology-related Variables in Specific Left Atrial Appendage (LAA) Morphology Types in the Main Stroke Group (n = 111).

LAA morphology	n (%)	Age, years	LAA volume unadjusted, mL	LAA volume adjusted for height, mL/m	LAA volume adjusted for body surface area, mL/m2	Enlarged LAA volume adjusted for body surface area
		Mean ± SD	Mean ± SD	Mean ± SD	Mean ± SD	n (% of total morphology type)
Cactus	10 (9.0)	62.6 ± 8.7	12.4 ± 5.7	7.2 ± 3.3	6.5 ± 3.2	6 (60)
ChickenWing	26 (23.4)	56.5 ± 12.0	13.9 ± 6.0	8.1 ± 3.3	7.1 ± 2.9	16 (62)
WindSock	53 (47.7)	61.9 ± 11.4	13.5 ± 4.8^a^	7.9 ± 2.8^a^	6.9 ± 2.5^a^	41 (77)
CauliFlower	22 (19.8)	60.6 ± 10.3	9.0 ± 3.2^b^	5.2 ± 1.9^b^	4.6 ± 1.8^b^	6 (27)
**LAA morphology**	**n (%)**	**Age, years**	**LAA volume unadjusted, mL**	**LAA volume adjusted for height, mL/m**	**LAA volume adjusted for body surface area, mL/m2**	**Enlarged LAA volume adjusted for body surface area**
		Mean ± SD	Mean ± SD	Mean ± SD	Mean ± SD	n (% of total morphology type)
Cactus	10 (9.0)	62.6 ± 8.7	12.4 ± 5.7	7.2 ± 3.3	6.5 ± 3.2	6 (60)
ChickenWing	26 (23.4)	56.5 ± 12.0	13.9 ± 6.0	8.1 ± 3.3	7.1 ± 2.9	16 (62)
WindSock	53 (47.7)	61.9 ± 11.4	13.5 ± 4.8^a^	7.9 ± 2.8^a^	6.9 ± 2.5^a^	41 (77)
CauliFlower	22 (19.8)	60.6 ± 10.3	9.0 ± 3.2^b^	5.2 ± 1.9^b^	4.6 ± 1.8^b^	6 (27)

Note: Significance assessed using the Mann-Whitney U-test

^a^ *P*<0.05

^b^ *P*<0.001

SD = standard deviation

There was a significant difference regarding obesity, BSA and hypertension between stroke and healthy controls. To test the possible confounding effect of hypertension and obesity, the 111 stroke patients were further divided into subgroups (hypertensive versus non-hypertensive and obese versus non-obese using BMI 30 kg/m^2^ as a cut-off). There was no statistical difference in the prevalence of LAA morphology types between these subgroups (*P* = 0.587 for hypertension, and *P* = 0.903 for obesity, respectively). Morphology types did neither exhibit any association with other baseline characteristics listed in Table 1.

Reproducibility analysis for LAA morphology with 40 randomly chosen stroke patients indicated good reproducibility (Cohen’s kappa value of 0.65 for inter-observer reproducibility and 0.67 for intra-observer reproducibility). There were no morphological differences (Cohen’s kappa value of 1) between arterial and venous phase in any of these patients.

## Discussion

The objective of the current study was to compare LAA morphology between patients with acute ischemic stroke of cryptogenic or suspected cardiogenic etiology other than known AF and control subjects without any cardiovascular disease. The prevalence of individual morphology types (Cactus, ChickenWing, WindSock, or CauliFlower) differed significantly between stroke patients and controls; stroke patients had a significantly larger proportion of single-lobed LAAs and larger LAA volumes.

Over 90% of the intracardiac thrombi in patients with cardioembolic stroke/TIA originally develop in the LAA [3]. Morphological and quantitative features of LAA have been increasingly studied, especially after the introduction of closing procedures as an alternative to anticoagulation in the treatment of AF patients [12]. Wang et al [12] were the first to present the division of LAA morphology into four different classes based on subjective visual criteria. Di Biase et al [16] applied this classification and correlated LAA morphology with the history of stroke events. Based on the same classification model adjusted for quantitative measurements, Kimura et al [13] allowed greater objectivity and reproducibility. Despite these attempts, the association between LAA morphological features and increased stroke risk has not been straightforward, and conflicting results have been published [17].

### LAA Morphology

Combining visual and quantitative methods, LAA morphology differed significantly between stroke patients and controls in the present study. There were no differences in morphological classifications between the arterial and venous phases.

Table 3 summarizes the methods and main findings of other stroke or stroke risk-related studies on LAA morphology over recent years. Our results parallel the studies of Di Biase, Kimura, and Yamamoto, suggesting a correlation between certain LAA morphologies and increased stroke risk [13,16,18], contradicting the studies of Khurram and Koplay [17,21]. Our analysis found a significantly increased prevalence of ChickenWing morphology in the matched stroke subgroup compared to the control group. This finding is in line with Kimura's result. However, in Di Biase’s study, non-ChickenWing was the most prevalent morphology type in stroke patients, whereas Anselmino’s study suggested an association between CauliFlower and WindSock morphologies and silent cerebral ischemia [16,19].

**Table 3:** Left Atrial Appendage (LAA) Morphology and Increased Stroke Risk in the Literature.

Group, year	Total N (Males, N [%])	Mean age ± SD	Modality	Indication for imaging	Type of study	LAA classification (number of classes)	Stroke- associated LAA morphology	Other findings
Yamamoto et al, 2014 [18]	564 (457[81%])	61 ± 11	TEE (3D)	AF ablation	Prospective	Complex vs. non-complex morphology (2)	Complex morphology	Increased number of LAA lobes independently associated with LAA thrombus formation
Kimura et al, 2013 [13]	80 (66[83%])	59 ± 6	CT	AF ablation	Retrospective	Quantitative Wang’s model (4)^a^	ChickenWing	CauliFlower dominant morphology (40%)
Khurram et al, 2013 [17]	678 (507[75%])	60 ± 10	CT	AF ablation	Retrospective	Wang’s model (4)^b^	Different morphologies not associated with stroke	ChickenWing dominant morphology (58%); short LAA length associated with stroke
Anselmino et al, 2013 [19]	348 (274[79%])	57 ± 11	CT/MRI	AF ablation and SCI burden assessment	Retrospective	Wang’s model (4)	NA	CauliFlower and Windsock associated with the prevalence of SCI
Makino et al, 2013 [20]	103 (NA)	NA	TEE and cCT	AF	Retrospective	Wang’s model (4)	NA	ChickenWing morphology associated with elevated LAA flow velocity
Park et al, 2013 [8]	264 (212[80%])	55 ± 11	3D-CT and TEE	AF ablation	Retrospective	No classification, only quantitative LAA measurements	NA	Depressed systolic function of the LAA was significantly related to stroke/TIA and recurrence of AF after catheter ablation
Di Biase et al, 2012 [16]	932 (734[79%])	59 ± 10	CT/MRI	AF ablation	Retrospective	Wang’s model (4)	Non-ChickenWing	ChickenWing dominant morphology (48%); Windsock more common in men
Koplay et al, 2012 [21]	320 (223[70%])	58 ± NA	MDCT	Various indications for CAD	Retrospective	Modified Lacomis’ model (5)^c^	Different morphologies not associated with thrombus formation	Dominant morphology was type 2b (Fan) where LAA tip was short, not prominent and inferiorly oriented
Shi et al, 2012 [22]	75 (42[56%])	42 ± 12	CTA	AF or ASD	Prospective	Shi’s model (8)^d^	NA	Tube morphology with a single lobe was more common in AF patients than ASD patients
Walker et al, 2012 [23]	59 (43[73%])	60 ± 8	CT	AF ablation	Prospective	No classification, only quantitative LAA measurements	NA	Strong correlation between increased LAA volume and LA volume; LAA orifice dimensions were significantly larger in persistent AF than PAF

^a^ According to LAA quantitative measurements: Cactus, ChickenWing, CauliFlower, WindSock

^b^ According to LAA external appearance: Cactus, ChickenWing, CauliFlower, WindSock

^c^ According to LAA tip orientation and external appearance: Horseshoe, Hand-finger, Fan, Wing, Hook, Wedge, Swan

^d^ According to LAA external appearance: Tube, Claw, Sphere-like, Tadpole, Willow-leaf, Sword, Duckbill, Irregular

AF = atrial fibrillation; ASD = atrial septal defect; CAD = coronary artery disease; cCT = cardiac CT; CT = computed tomography; CTA = CT angiography; LAA = left atrial appendage; MDCT = multidetector CT; MRI = magnetic resonance imaging; NA = not applicable; PAF = paroxysmal AF; SCI = silent cerebral ischemia; SD = standard deviation; TEE = transesophageal echocardiogram

Conflicting results regarding LAA morphology may be explained, in part, by different classification criteria, and by variation in and overlap of morphological classes. Concensus regarding the most valid classification is lacking, and all of the current classifications may be too arbitrary. Our approach to LAA morphological analysis was visual and consensus-based, obviating time-consuming 3D reconstructions from the original CT scans, which makes this method clinically more feasible. Notably, we used Kimura’s quantitative qualifier alongside the visual analysis in our study. In most recent studies, morphological agreement was based purely on visual analysis. In addition, patient selection varies between studies. AF was an inclusion criterion in almost all previous studies on LAA morphology and stroke, but our study excluded all patients with known AF. To the best of our knowledge, we are the first to study LAA morphology in a prospectively collected stroke population without carotid artery atherosclerosis or known AF. In this group, exploring stroke mechanism is demanding and new clinical tools are needed. Multiple CT scans were not performed during the follow-up, which would have enabled observation of long-term changes in LAA morphology and its possible relations to pathological conditions, such as to hypertension.

### LAA Lobes and Volume

The other main finding in our study was the significantly higher proportion of single-lobed LAAs in the matched stroke subgroup compared to the control group. In addition, LAA volume was significantly larger in the matched stroke subgroup. Based on these findings it can be hypothesized that the increase of LAA volume and the decrease of the number of LAA lobes might be consequences of a pathological remodeling process [24–25]. It is known that cardiac diseases, such as left ventricular hypertrophy or mitral valve regurgitation, change the hemodynamics and volume of both the LA and LAA [26]. Thus, fewer LAA lobes might represent a phenomenon secondary to a pathological condition.

Alterations in the LAA wall and surrounding structures also offer a possible pathophysiological explanation for elevated LAA volumes in stroke patients. A previous study indicated an association between LAA volume and pericardial adipose tissue (PAT) area [27]. A lack of supporting tissue around the LAA wall may predispose an individual to more rapid loosening of normal wall structures and result in enlargement and bending of the LAA body. Bending might modify the blood current inside the LAA, cause turbulence and result in thrombus formation over time. If this is the case, large and single-lobed morphologies could be more prone to thrombosis.

In contrast to the above hypothesis, some previous studies suggest that extensive LAA trabeculation and multiple lobes, like in CauliFlower morphology type, cause poor emptying and slow blood filling in the LAA increasing the risk of stroke/TIA [17–18,28]. Yamamoto’s result supports this theory, as the most trabeculated morphology presented an independent risk for stroke [18]. In addition, decreased LAA length may be an independent risk factor for stroke [17].

From a clinical point of view, distinguishing patients with enlarged LAA and high-risk LAA morphology might aid in etiological workup and secondary prevention of stroke, even in the absence of direct evidence of intracardiac thrombus or AF at the time of evaluation. Our results parallel the fact that extending rhythm monitoring beyond 24-h ECG Holter is reasonable to detect long-interval paroxysmal AF in patients with high-risk LAA factors. In the future, cardiac imaging will become more frequent in the assessment of stroke etiology due to low-dose scanners. However, more studies are needed to identify which, if any, of the morphological LAA features is a clinically significant risk factor for stroke.

The main limitation was that the number of patients was relatively small. The study population included stroke/TIA patients with clinically suspected cardioembolic origin without previously known AF or AF in immediate post-stroke 24-h Holter ECG. However, not all PAF may be revealed during 24-h Holter ECG [29]. Moreover, our study patients were not followed beyond three months, and thus the incidence of late-onset atrial fibrillation, thrombi, or recurrent strokes is not known. In addition, the LAA morphology is not established, and the classification used in our study may still be oversimplifying when associations to stroke are examined.

## Conclusions

The LAA morphology in stroke/TIA patients without known AF seems to differ from that of healthy individuals. Single-lobed and large LAAs seem to be over-represented among stroke/TIA patients. Future studies are needed to evaluate whether these changes have an impact on thrombus formation in the LAA.
